# Microhemorrhages in MELAS Lesions: A Case Report

**DOI:** 10.5334/jbsr.2891

**Published:** 2022-09-30

**Authors:** Evelien Martens, Jelle Demeestere, Benjamin Verhaaren

**Affiliations:** 1 UZLeuven, BE

**Keywords:** MELAS, MRI, microbleeds, microhemorrhages, stroke-like episodes

## Abstract

**Introduction:**

Microhemorrhages have not been described in mitochondrial encephalomyopathy with lactic acidosis and stroke-like episodes syndrome (MELAS) on magnetic resonance imaging (MRI). Main symptoms and/or important findings: A MELAS-patient had a rapid succession of 3 stroke-like episodes with dysphasia, visual field deficits and paresis of the right arm. MRI showed a lesion with corticosubcortical vasogenic edema without reduced diffusion, conforming to a stroke-like MELAS-lesion. Microhemorrhages within MELAS-lesions were detected on MRI. The main diagnoses, therapeutic interventions, and outcomes: Microhemorrhages are an atypical imaging finding in MELAS. The patient was treated with L-arginine.

**Conclusion:**

Microhemorrhages can present on MRI in (sub)acute MELAS lesions and may reflect mitochondrial microangiopathy.

## Introduction

Mitochondrial encephalomyopathy with lactic acidosis and stroke-like episodes syndrome (MELAS) is a rare genetic mitochondrial disease that may cause stroke-like episodes, epilepsy, recurrent headaches, and dementia [1]. Typical magnetic resonance imaging (MRI) findings include gyral swelling with a predilection for the parieto-occipital and parieto-temporal regions with T2/Fluid-attenuated inversion recovery (FLAIR) hyperintensity and increased signal of cortices on diffusion-weighted imaging (DWI), with normal or low apparent diffusion coefficient (ADC) values [2,3].

## Case History

A 35-year-old male with a previously established diagnosis of MELAS with a point mutation at nucleotide 3243 mtDNA (A>G) presented with aphasia for two days, behavior changes for two months, and subtle loss of strength in the right arm on clinical examination.

**Figure 1:**
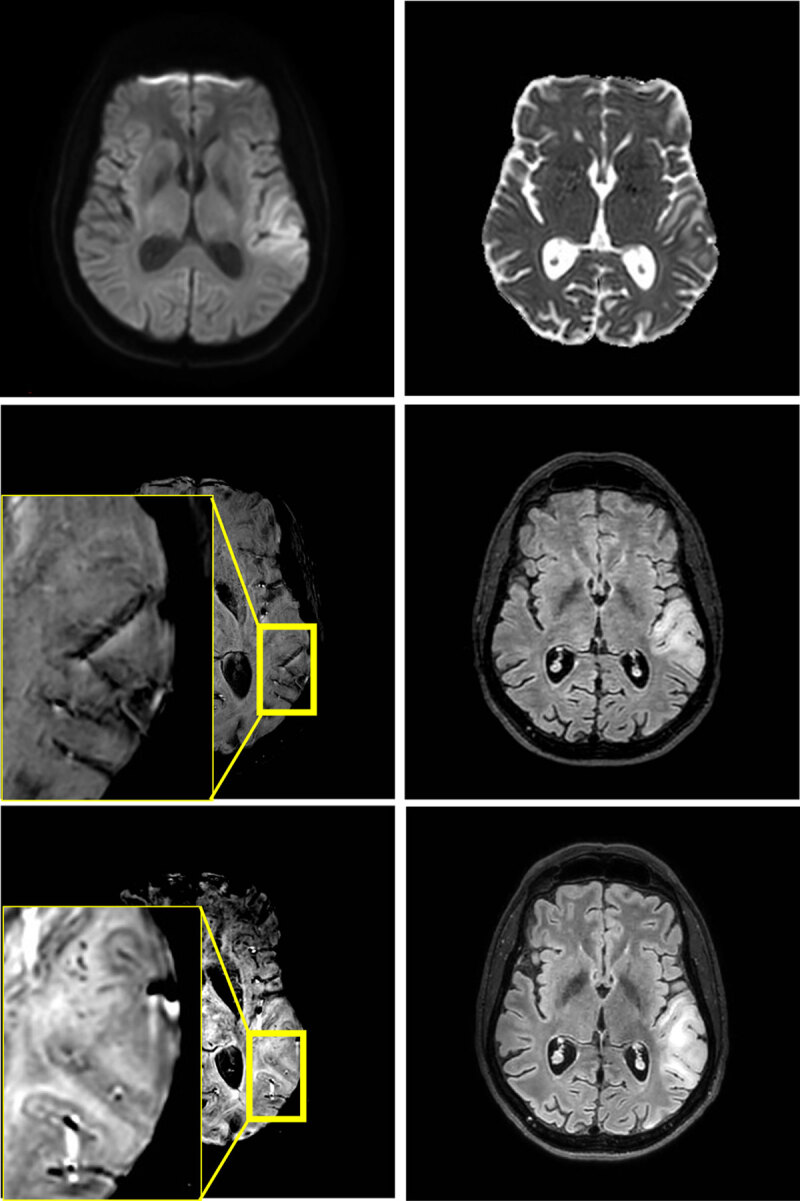
Edema in the left temporoparietal operculum without reduced diffusivity on MRI at 3T at first presentation (4 upper images). No microhemorrhages are found on the first scan, but they are present in juxtacortical location at one-week follow-up (2 lower images).

An immediate computed tomography (CT) and CT angiography (CTA, Siemens) showed corticosubcortical hypodensity in the left temporoparietal operculum, and normal craniocervical arteries. The next day’s MRI (3 Tesla, Philips) showed local corticosubcortical vasogenic edema without reduced diffusion, in keeping with a stroke-like MELAS lesion (Figure 1). Treatment with L-arginine was initiated, and the aphasia improved during hospitalization. A follow-up MRI (3 Tesla, Philips) after one week showed further expansion of the lesion that now demonstrated reduced diffusion as well as multiple, local, juxtacortical microhemorrhages on susceptibility weighted imaging (SWI), of which most were remarkably fine (< 1 mm).

**Figure 2:**
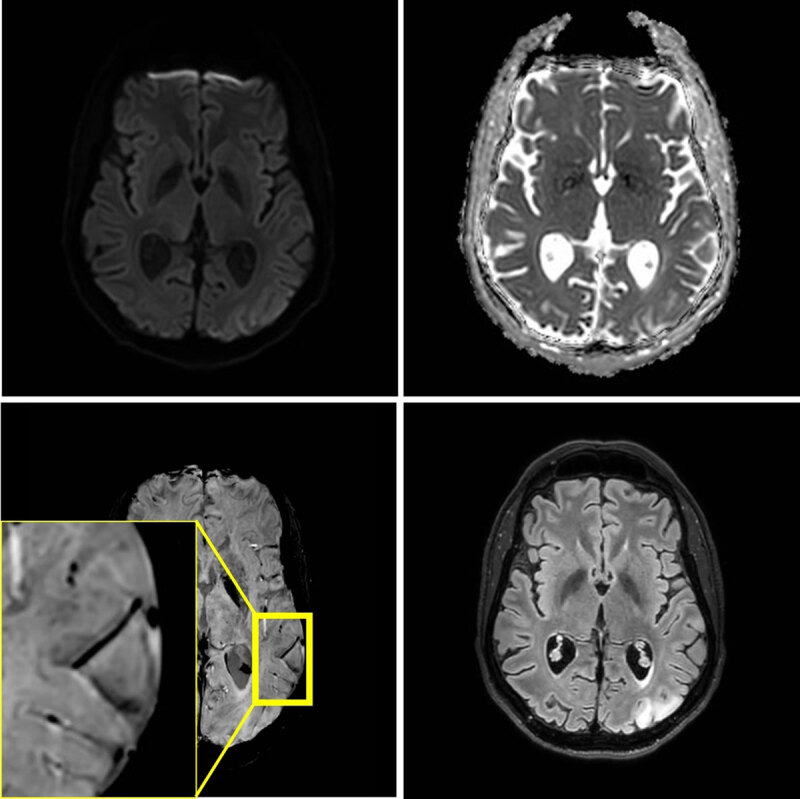
MRI at 3T performed three weeks later, because of a second stroke-like event. A similar lesion in the gyri of the left occipital lobe is present. Signal abnormalities in the previously affected area nearly normalized, but microhemorrhages persisted.

**Figure 3:**
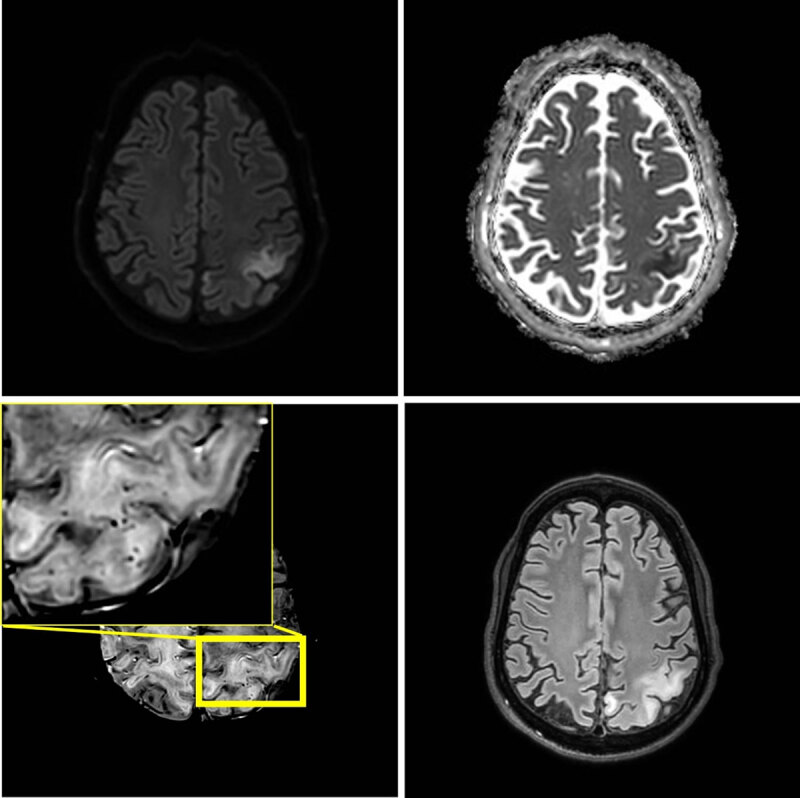
One month later, seven weeks after the initial episode, a third stroke-like event occurred. MRI shows a new area of edema in the left parietal lobe including the postcentral gyrus. The new lesion shows diffusion restriction and juxtacortical microhemorrhages.

After three and seven weeks, two new stroke like-episodes occurred with similar imaging findings respectively in the left occipital region with right hemianopia, and in the left parietal region including the postcentral gyrus with right arm paresis and sensory deficits (Figures 2 and 3). Notably, at seven weeks, microhemorrhages were also present in the acute lesion. Signal abnormalities in previously affected areas nearly normalized, but microhemorrhages persisted.

Eventually, the patient left the hospital in good general health and underwent further rehabilitation.

## Comment

To our knowledge this is the first report of a case of juxtacortical microhemorrhages in MELAS lesions on MRI. As findings were subtle, these may not have been detected with conventional T2*-sequences that were more often used in the past. True hemorrhagic transformation of a MELAS lesion has been reported, but is rare [4,5]. Our findings may suggest that this could be related to underlying mitochondrial microangiopathy. Further research is needed to assess the true incidence of microhemorrhages in MELAS and whether they increase the risk of macroscopic parenchymal hemorrhages.
